# The Effect of Intra- and Intergenerational Caregiving on Subjective Well-Being – Evidence of a Population Based Longitudinal Study among Older Adults in Germany

**DOI:** 10.1371/journal.pone.0148916

**Published:** 2016-02-09

**Authors:** André Hajek, Hans-Helmut König

**Affiliations:** Department of Health Economics and Health Services Research, Hamburg Center for Health Economics, University Medical Center Hamburg-Eppendorf, Hamburg, Germany; Federal University of Rio de Janeiro, BRAZIL

## Abstract

**Objective:**

To examine whether intra- and intergenerational caregiving affect subjective well-being (SWB) of the caregivers longitudinally.

**Methods:**

Data were drawn from the German Ageing Survey (DEAS), which is a population-based longitudinal study of individuals living in Germany aged 40 and over. The waves in 2002, 2008 and 2011 were used (with 10,434 observations). SWB was examined in a broad sense, covering affective (AWB) and cognitive well-being (CWB), positive (PA) and negative affect (NA) as well as functional and mental health. While intragenerational caregiving was defined as providing care for spouse/partner, intergenerational caregiving was defined as providing care for mother, father, mother-in-law, father-in-law, partner’s mother or partner’s father.

**Results:**

Fixed effects regressions adjusting for sociodemographic factors, social network, self-efficacy and morbidity showed that intergenerational informal care did not affect the various SWB outcome measures. Intragenerational caregiving affected CWB (women) and mental health (total sample and men), whereas it did not affect the other outcome variables.

**Conclusion:**

Our findings highlight the importance of intragenerational caregiving for mental health (men) and cognitive well-being (women). Consequently, interventions to avoid mental illness due to intragenerational caregiving are urgently needed.

## Introduction

Subjective well-being (SWB) refers to the numerous ways in which people evaluate the quality of their lives [1]. It is a wide concept with two main components, cognitive well-being (CWB) and affective well-being (AWB) [2]. CWB refers to the cognitive evaluation of one’s life, whereas AWB refers to the experience of positive affects (PA) including joy or activation and the absence of negative affects (NA) such as sadness or fear [3]. AWB and CWB should be seen as different constructs since they differ in their predictors [4] and their long-term stability [5]. Moreover, similar concepts exist in medical research, though with a stronger focus on *health*-related quality of life (HRQoL). HRQoL includes the core components functional and mental health and is one of the most important health outcome measures [6, 7].

It is well-known that informal caregiving is one of the main predictors of HRQoL cross-sectionally [8, 9]. According to the social exchange theory [10], to what degree informal caregiving affects SWB is influenced by the quality of our relationships, i. e. whether care is provided for the partner (*intra*generational) or the mother(-in-law)/father(in-law) (*inter*generational). Especially transitions to spousal caregiving are associated with adverse health outcomes in caregivers [11]. In most cases, the risk for onset of distress is highest in individuals starting to provide care for a spouse or partner, with higher values in women [12]. The great strain in spousal caregivers was confirmed in previous studies [13–15]. This can be explained by the fact that compared with other informal care settings spouses tend to provide more hours of caregiving, showed a higher responsibility for caregiving tasks and experience greater emotional closeness with the care-recipients [16]. In contrast, parental caregiving by adult children might provide more time and space for rest and recreation. Nevertheless, even daughters or sons caring for their parents(in-law) might experience outcomes such as anxiety, frustration, disruption of their lives or a lack of free time [17, 18]. Therefore, alterations in caregivers’ well-being might result in misconduct including abusive behavior [19–21].

Nonetheless, care recipients prefer to live at home as long as possible for reasons of keeping their social relations and to maintain their surrounding environment. By considering these preferences, it is most likely that the need for informal care will increase considerably in the next decades due to demographic shifts, which underlines the relevance of this issue.

To our knowledge, longitudinal studies are missing investigating the long-term effect of intra- and intergenerational caregiving on SWB in a wide sense, covering AWB and CWB as well as HRQoL. Thus, in order to close this significant research gap we aimed at examining whether intra- and intergenerational caregiving affect the various measures of SWB differently in the long run, using a representative sample of community-dwelling adults aged 40 years and above in Germany. Thereby, the population at risk for worsening SWB can be identified. Furthermore, conclusions about the causal relationship between intra-/intergenerational caregiving and SWB can be drawn, which is important to develop new treatment strategies. Moreover, and contrary to cross-sectional regressions, by using fixed effects (FE) regressions (panel data method), time-constant unobserved heterogeneity can be taken into account, leading to consistent estimates.

## Methods

### Sample

Data were derived from the public release of the German Ageing Survey (DEAS), provided by the Research Data Centre of the German Centre of Gerontology (DZA) which is a population-based, representative (national probability sampling) survey of the community-dwelling population aged 40 and above in Germany.

Our analysis was restricted to the waves 2 to 4 as a measure of depression was included for the first time in the second wave. 5,194 individuals took part in the second wave, whereas 8,200 individuals took part in the third wave and 4,855 individuals took part in the fourth wave. Due to the introduction of new samples, sample sizes differed markedly between waves. Thereby, it is worth mentioning that while 6,205 (number of usable interviews in the third wave) community-dwelling individuals from the birth cohorts from 1923–1968 were interviewed for the first time in the third wave, 1,995 had already been interviewed in former waves. Engstler and Motel-Klingenbiel [22] provide more details concerning the sampling frame and the sample composition. Written informed consent was given prior to the interview.

### Outcome: Subjective well-being

CWB was quantified by using the Satisfaction with Life Scale (SWLS, [23]) which consists of five items on a five point rating scale (1–5, higher values indicate higher CWB). Additionally, PA and NA were measured by using the Positive and Negative Affect Schedule (PANAS, [24]), each with ten items on a five point rating scale, ranging from 1 (very slightly / not at all) to 5 (extremely). Therefore, high values indicate high positive or negative affect.

Mental health was quantified by using the Center for Epidemiologic Studies Depression Scale (CES-D, [25]), with 15 items (sum score 0–45, with higher values indicating worse ratings of mental health). Functional health was assessed by the subscale “physical functioning” of the 36-Item Short Form Health Survey (SF-36, ranging from 0 (worst) to 100 (best) [26]).

### Independent variables

The question “Are there people you look after or care for regularly due to their poor state of health, either on a private or volunteer basis?” (no; yes) was used to quantify informal caregiving. Thereafter informal caregivers were asked for whom they provide support (mother, father, mother-in-law, partner’s mother, father-in-law, partner’s father, spouse/partner, neighbors and numerous other options). Even though some other care relationships are also recorded in DEAS (such as acquaintances or own children), they occur too rarely for robust regression analysis. Consequently, intergenerational caregiving was defined as follows: providing care for mother, father, mother-in-law, father-in-law, partner’s mother or partner’s father. Intragenerational care was defined as follows: providing care for spouse/partner.

Moreover, time-dependent predictors (i.e. any predictor whose value for a given individual may change over time) assumed to be important for SWB were considered in regression analysis such as sociodemographic variables [27], social network, self-efficacy and morbidity [28]. Thus, age and (log) monthly household net income in Euro were included. Furthermore, the social network was quantified by using the number of important people in regular contact (from 0 to 9). Self-efficacy was assessed by the HOPE scale (four point rating scale, eight items), with higher values indicating higher self-efficacy. Additionally, morbidity was assessed by the total number of physical diseases, e. g. cancer, respiratory diseases or hearing problems, informed by the Charlson Comorbidity Index [29].

Moreover, dummy coded variables were included for employment status (Ref.: working; retired; other: not employed), region (states), and family status (Ref.: married, living together with spouse; married, living separated from spouse; divorced; widowed; never married). However, these variables were not shown in regression tables for the sake of space (but they are available upon request).

The time-constant independent variables education (quantified by the International Standard Classification of Education, ISCED [30] with three categories: low (ISCED 0–2), medium (ISCED 3–4) and high (ISCED 5–6)) and sex were depicted at baseline for descriptive purposes. These time-constant factors are excluded from regression analysis because solely time-dependent predictors can be included in FE regression (please see: next chapter).

### Statistical analysis

FE regressions were used to estimate the effect of time-dependent independent variables on SWB. This is important in order to take time-constant unobserved factors (e.g. genetic predisposition or personality) into account. This in turn is crucial because unobserved factors are frequently systematically correlated with independent variables in SWB research [31, 32]. For instance, cross-sectional regressions generally revealed that marriage and CWB are positively related. This can be explained by self-selection, meaning that individuals with higher CWB might select themselves into marriage. Longitudinal regressions might reveal that marriage did not affect CWB.

If—as it is usually the case—time-constant unobserved factors are correlated with the predictors, random effects regressions lead to inconsistent estimates, therefore FE regressions are the method of choice as they provide consistent estimates under the assumption of strict exogeneity [33]. FE regressions only use within-variations over time (i.e. intraindividual changes, e.g. not being a caregiver in wave 2 and being a caregiver in wave 3 and wave 4). For that reasons, the FE estimator is also called ‘within-estimator’ (for technical details: [33]). This is why only time-dependent variables can be included in FE regressions. Standard errors that cluster errors at the individual level were computed to take heteroscedasticity and serial correlation of the error terms into account [34].

Generally, a panel regression model can be written as

$$Y_{it}=\alpha_{i}+\beta X_{it}+\gamma_{i}W_{i}+\lambda_{t}+\varepsilon_{it}$$

i = 1, …, N: units (persons); t = 1,…, T: time

λ_t_ are factors changing over time, but are constant across individuals. Moreover, W_i_ are constant observed characteristics of individual units. The time-dependent outcome variable is denoted as Y_it_, time-dependent idiosyncratic errors are denoted as ε_it,_ and time-dependent covariates are denoted as X_it_.

In contrast to cross-sectional regressions, an individual specific intercept α_i_ is included. It captures the effect of unobserved time-constant factors of an individual i on outcomes Y_i_. It is of relevance when the model allows a correlation between observed independent variables and the parameter α_i_—addressing the endogenous selection into treatment (based on time-constant unobserved factors). This is achieved by the FE-estimator.

The FE-estimator uses within-transformed data (also called: change score or demeaned data) to estimate the equation mentioned above from variation in observed predictors and outcome variables (within individuals over time):

$$Y_{it}-{\bar{Y}}_{i}=\beta \left(X_{it}-{\bar{X}}_{i}\right){+\lambda }_{t}-\bar{\lambda }+\left(\varepsilon_{it}-{\bar{\varepsilon }}_{i}\right)$$

The effect of time-constant unobserved heterogeneity (unobserved α_i_ and observed W_i_) is eliminated by differencing the data. As a result, changes in outcomes (Y_it_-${\bar{\text{Y}}}_{\text{i}}$) only depend on changes in time-dependent covariates X_it_ and time-dependent idiosyncratic errors ε_it_.

Furthermore, it is worth mentioning that the Stata command for FE regression analysis include individuals with only one observation in calculating the number of observation as they provide information about the constant and the variance components and so on. However, it does not affect the standard errors and the beta-coefficients.

## Results

### Descriptive analysis

The majority was male (51.5%) and had according to ISCED categories a medium education (52.5%) at wave 2. Descriptive statistics were reported in Table 1 for time-dependent variables (in individuals reporting SWB outcomes in at least two of the three waves since only within information can be included in FE regression analysis). The mean age was 59.1 years (±10.4 years, 40–83 years) at wave 2. Most of them were still working (44.7%) and were married, living together with spouse (78.0%). The mean number of important people in regular contact was 5.2 (±2.5), mean self-efficacy was 3.1 (±0.4) and the mean number of physical diseases was 2.2 (±1.7). While most people did not provide informal care (90.6%), some individuals provide inter- (7.3%) or intragenerational (2.1%) care.

**Table 1:** Descriptive statistics for time-dependent variables over time (Waves 2–4).

	Wave 2 (n = 1,646)	Wave 3 (n = 3,046)	Wave 4 (n = 3,023)
Age: Mean (SD)	59.1 (10.4)	63.1 (11.1)	65.5 (10.7)
Marital status^1^: N (%)			
Married, living together with spouse	1,283 (78.0)	2,249 (73.8)	2,227 (73.7)
Married, living separated from spouse	25 (1.6)	40 (1.3)	34 (1.1)
Divorced	134 (8.1)	254 (8.4)	249 (8.3)
Widowed	134 (8.1)	345 (11.3)	355 (11.8)
Single	69 (4.2)	158 (5.2)	155 (5.1)
Employment status^2^: N (%)			
Working	736 (44.7)	1,082 (35.5)	958 (31.7)
Retired	669 (40.6)	1,599 (52.5)	1,752 (58.0)
Other: not employed	241 (14.7)	365 (12.0)	309 (10.3)
Monthly household net income in Euro^3^: Mean (SD)	2,987.9 (1875.3)	2,587.8 (2447.6)	2,714.5 (1715.0)
Number of important people in regular contact^4^: Mean (SD)	5.2 (2.5)	4.7 (2.8)	5.0 (2.7)
Self-efficacy (HOPE Scale)^5^: Mean (SD)	3.1 (0.4)	3.0 (0.4)	3.0 (0.4)
Morbidity (total number of physical diseases)^6^: Mean (SD)	2.2 (1.7)	2.4 (1.8)	2.6 (1.9)
Informal care^7^: N (%)			
Not providing informal care	1,416 (90.6)	2,512 (90.0)	2,481 (89.5)
Providing intergenerational informal care	114 (7.3)	205 (7.3)	195 (7.0)
Providing intragenerational informal care	33 (2.1)	75 (2.7)	95 (3.4)
Functional health (Subscale 'Physical Functioning' of the SF-36)^8^: Mean (SD)	88.5 (17.0)	84.9 (19.8)	82.7 (21.3)
Mental Health (CES-D)^9^: Mean (SD)	7.1 (5.9)	6.5 (5.4)	7.0 (5.8)
CWB (SWLS)^10^: Mean (SD)	3.9 (0.7)	3.8 (0.7)	3.8 (0.7)
NA (PANAS)^11^: Mean (SD)	2.0 (0.5)	2.1 (0.5)	2.1 (0.5)
PA (PANAS)^12^: Mean (SD)	3.5 (0.5)	3.5 (0.5)	3.5 (0.5)

Missing values for all variables (if occurred)

^1^ 1 missing value in the second wave and 3 missing values in the fourth wave

^2^ 4 missing values in the fourth wave

^3^ 435 missing values in the second wave, 445 missing values in the third wave and 261 missing values in the fourth wave

^4^ 17 missing values in the second wave, 1 missing value in the third wave

^5^ 41 missing values in the second wave, 90 missing values in the third wave and 100 missing values in the fourth wave

^6^ 43 missing values in the second wave, 128 missing values in the third wave and 144 missing values in the fourth wave

^7^ 83 missing values in the second wave; 252 missing values in the third wave; 252 missing values in the fourth wave

^8^ 17 missing value in the second wave, 3 missing values in the third wave and 13 missing values in the fourth wave

^9^ 91 missing values in the second wave, 89 missing values in the third wave and 65 missing values in the fourth wave

^10^ 42 missing values in the second wave, 90 missing values in the third wave and 104 missing values in the fourth wave

^11^ 43 missing values in the second wave, 91 missing values in the third wave and 102 missing values in the third wave

^12^ 43 missing values in the second wave, 91 missing values in the third wave and 102 missing values in the third wave; SD: Standard deviation

Mean CWB (SWLS) was 3.9 (±0.7), mean NA (PANAS) was 2.0 (±0.5), mean PA (PANAS) was 3.5 (±0.5), mean functional health (Subscale ‘Physical Functioning‘ of the SF-36) was 88.5 (±17.0) and mean mental health (CES-D) was 7.1 (±5.9). While the proportion of employed individuals decreased markedly after 9 years, the other variables remained almost the same.

### Regression analysis

FE regressions (with 10,434 observations (NA as outcome variable), n_first wave_ = 2,986, n_second wave_ = 4,356, n_third_ wave = 3,092) revealed that intergenerational caregiving did not affect outcome variables in the total sample and in both sexes (Table 2). While intragenerational caregiving also did not affect NA, PA and functional health in the total sample and in both sexes, intragenerational caregiving affected CWB (β = -.2) in women (Table 3). Furthermore, intragenerational caregiving affected mental health in the total sample (β = 3.0) and in men (β = 4.2).

**Table 2:** Intergenerational Caregiving—Longitudinal predictors of SWB (CWB: cognitive well-being; NA: negative affect: PA: positive affect): Results of fixed effects regressions (Waves 2–4).

VARIABLES	CWB	CWB—Men	CWB—Women	NA	NA—Men	NA—Women	PA	PA—Men	PA—Women	Functional health	Functional health—Men	Functional health—Women	Mental health	Mental health—Men	Mental health—Women
Age	0.0124***	0.0117***	0.0140***	-0.00913***	-0.00818**	-0.00984**	0.00336+	-0.000191	0.00798**	-0.576***	-0.541***	-0.588***	-0.106***	-0.0872**	-0.129**
	(0.00238)	(0.00317)	(0.00359)	(0.00207)	(0.00272)	(0.00320)	(0.00183)	(0.00239)	(0.00282)	(0.0681)	(0.0949)	(0.0988)	(0.0253)	(0.0300)	(0.0426)
Morbidity	-0.0371***	-0.0370***	-0.0347**	0.0734***	0.0703***	0.0787***	-0.0236***	-0.0177*	-0.0286**	-1.213***	-1.468***	-0.940**	0.369***	0.355***	0.398**
	(0.00731)	(0.00942)	(0.0112)	(0.00665)	(0.00877)	(0.0103)	(0.00579)	(0.00717)	(0.00943)	(0.212)	(0.296)	(0.296)	(0.0801)	(0.105)	(0.126)
Monthly household net income in Euro (log)	0.114***	0.0701+	0.148**	0.0172	0.0160	0.0148	0.0353	0.00963	0.0624+	-0.498	1.067	-2.113	-0.149	-0.368	0.148
	(0.0323)	(0.0414)	(0.0498)	(0.0278)	(0.0390)	(0.0399)	(0.0256)	(0.0370)	(0.0361)	(1.096)	(1.183)	(1.879)	(0.342)	(0.428)	(0.537)
Number of important people in regular contact	-0.000148	0.000634	-0.00184	0.00507+	0.00417	0.00681	0.00886***	0.00982**	0.00699+	-0.153+	-0.110	-0.183	-0.0719*	-0.0424	-0.106+
	(0.00305)	(0.00387)	(0.00485)	(0.00283)	(0.00377)	(0.00430)	(0.00247)	(0.00327)	(0.00379)	(0.0884)	(0.118)	(0.131)	(0.0339)	(0.0405)	(0.0572)
Self-efficacy	0.719***	0.748***	0.699***	-0.310***	-0.320***	-0.298***	0.470***	0.463***	0.488***	3.483***	3.733**	3.340**	-1.693***	-2.083***	-1.289*
	(0.0346)	(0.0459)	(0.0524)	(0.0303)	(0.0422)	(0.0444)	(0.0275)	(0.0389)	(0.0392)	(0.863)	(1.217)	(1.203)	(0.348)	(0.475)	(0.503)
Intergenerational informal caregiving (Ref.: no)	-0.0329	0.0473	-0.0925+	-0.0195	-0.0260	-0.0146	-4.23e-05	-0.0100	0.00704	0.509	0.0189	0.785	0.583	0.0848	0.985+
	(0.0352)	(0.0507)	(0.0480)	(0.0264)	(0.0431)	(0.0338)	(0.0249)	(0.0393)	(0.0323)	(1.005)	(1.596)	(1.290)	(0.378)	(0.426)	(0.578)
Constant	-0.627	-0.378	-0.168	2.944***	2.831***	3.525***	1.146**	1.512**	0.925*	127.6***	113.0***	127.1***	19.70***	18.01***	19.45***
	(0.494)	(0.585)	(0.456)	(0.308)	(0.393)	(0.410)	(0.434)	(0.509)	(0.367)	(10.51)	(11.75)	(16.70)	(3.912)	(4.436)	(5.429)
Observations	10,433	5,516	4,911	10,434	5,519	4,909	10,432	5,517	4,909	127.6***	113.0***	127.1***	10,248	5,417	4,825
Number of Individuals	7,511	3,921	3,587	7,511	3,924	3,584	7,509	3,922	3,584	(10.51)	(11.75)	(16.70)	7,388	3,855	3,530
R^2^	0.212	0.236	0.200	0.120	0.124	0.124	0.162	0.165	0.169	0.0758	0.0992	0.0639	0.0388	0.0464	0.0472

Comments: Beta-Coefficients were reported; Cluster-robust standard errors in parentheses

*** p<0.001

** p<0.01

* p<0.05

+ p<0.10

Regressions are also controlled for family status, employment status and region. Observations with missing values were dropped (listwise deletion). CWB (SWLS, higher values indicate high CWB); NA (PANAS, high values indicate high NA); PA (PANAS, high values indicate high PA); Functional health (Subscale 'Physical Functioning' of the SF-36, high values indicate high functional health); Mental health (CES-D, high values indicate low mental health).

**Table 3:** Intragenerational Caregiving—Longitudinal predictors of SWB (CWB: cognitive well-being; NA: negative affect: PA: positive affect): Results of fixed effects regressions (Waves 2–4).

VARIABLES	CWB	CWB—Men	CWB—Women	NAupp	NA—Men	NA—Women	PA	PA—Men	PA—Women	Functional health	Functional health—Men	Functional health—Women	Mental health	Mental health—Men	Mental health—Women
Age	0.0126***	0.0118***	0.0141***	-0.00867***	-0.00798**	-0.00916**	0.00261	-0.00153	0.00799*	-0.627***	-0.634***	-0.598***	-0.0913***	-0.0834*	-0.105*
	(0.00252)	(0.00332)	(0.00386)	(0.00220)	(0.00284)	(0.00350)	(0.00198)	(0.00255)	(0.00311)	(0.0711)	(0.0978)	(0.105)	(0.0266)	(0.0324)	(0.0446)
Morbidity	-0.0355***	-0.0319***	-0.0381**	0.0725***	0.0686***	0.0794***	-0.0210***	-0.0162*	-0.0258*	-1.413***	-1.640***	-1.142***	0.354***	0.371***	0.328*
	(0.00754)	(0.00953)	(0.0118)	(0.00667)	(0.00867)	(0.0106)	(0.00620)	(0.00748)	(0.0105)	(0.223)	(0.309)	(0.308)	(0.0803)	(0.101)	(0.131)
Monthly household net income in Euro (log)	0.123***	0.103*	0.140**	0.0216	-0.00192	0.0461	0.0449+	0.0123	0.0831*	-1.203	0.918	-3.643+	-0.300	-0.479	-0.0210
	(0.0333)	(0.0425)	(0.0526)	(0.0294)	(0.0397)	(0.0447)	(0.0272)	(0.0379)	(0.0404)	(1.204)	(1.266)	(2.116)	(0.363)	(0.449)	(0.583)
Number of important people in regular contact	-0.000959	0.00153	-0.00588	0.00496+	0.00420	0.00660	0.00733**	0.00758*	0.00590	-0.136	-0.0740	-0.197	-0.0518	-0.0101	-0.107+
	(0.00317)	(0.00391)	(0.00523)	(0.00295)	(0.00381)	(0.00470)	(0.00260)	(0.00332)	(0.00421)	(0.0940)	(0.120)	(0.147)	(0.0354)	(0.0421)	(0.0606)
Self-efficacy	0.718***	0.755***	0.684***	-0.306***	-0.312***	-0.299***	0.480***	0.471***	0.499***	3.704***	4.013***	3.495**	-1.471***	-1.979***	-0.978*
	(0.0357)	(0.0457)	(0.0558)	(0.0308)	(0.0421)	(0.0459)	(0.0291)	(0.0396)	(0.0434)	(0.858)	(1.172)	(1.245)	(0.333)	(0.469)	(0.474)
Intragenerational informal caregiving (Ref.: no)	-0.103+	-0.0269	-0.218*	0.0221	0.0130	0.0304	-0.0143	0.0221	-0.0440	-2.645	-3.797	-0.179	3.025***	4.240***	1.482
	(0.0577)	(0.0730)	(0.0928)	(0.0484)	(0.0592)	(0.0834)	(0.0550)	(0.0664)	(0.0940)	(2.148)	(2.573)	(3.616)	(0.697)	(0.855)	(1.124)
Constant	-1.060	-0.711	-0.0808	2.703***	2.999***	3.236***	0.655	1.498**	0.728+	126.9***	116.3***	140.0***	17.47***	18.06***	17.85**
	(0.684)	(0.595)	(0.478)	(0.346)	(0.399)	(0.440)	(0.623)	(0.524)	(0.414)	(11.69)	(12.34)	(18.29)	(4.356)	(4.658)	(5.814)
Observations	10,089	5,442	4,642	10,090	5,445	4,640	10,088	5,443	4,640	10,087	5,444	4,638	9,894	5,337	4,552
Number of Individuals	7,370	3,903	3,465	7,371	3,906	3,463	7,369	3,904	3,463	7,363	3,902	3,459	7,240	3,834	3,404
R^2^	0.219	0.243	0.202	0.115	0.121	0.120	0.163	0.166	0.170	0.0873	0.119	0.0677	0.0388	0.0601	0.0374

Comments: Beta-Coefficients were reported; Cluster-robust standard errors in parentheses

*** p<0.001

** p<0.01

* p<0.05

+ p<0.10

Regressions are also controlled for family status, employment status and region. Observations with missing values were dropped (listwise deletion). CWB (SWLS, higher values indicate high CWB); NA (PANAS, high values indicate high NA); PA (PANAS, high values indicate high PA); Functional health (Subscale 'Physical Functioning' of the SF-36, high values indicate high functional health); Mental health (CES-D, high values indicate low mental health).

In addition, longitudinal regressions showed that age (except for PA in the total sample and in men), morbidity and self-efficacy influenced each outcome measure in the total sample and in both sexes.

### Sensitivity analysis

The robustness–in terms of significance–of our findings was tested by comparing our main findings (Table 2 and Table 3) with an alternate model specification; i. e. in sensitivity analysis, intergenerational caregiving was restricted to parental caregiving (mother or father). Providing care for mother or father did not affect the outcome variables in the total sample and in both sexes, underlining our main findings (results are not shown, but are available upon request from the authors).

## Discussion

### Main findings

Longitudinal regressions showed that intergenerational caregiving did not affect the various SWB outcome measures. Intragenerational caregiving affected CWB (women) and mental health (total sample and men), whereas it did not affect the other outcome variables.

### Previous research

Transitions to spousal caregiving markedly increased stress levels in previous studies [13–15]. Nevertheless, the effect of intra- and intergenerational on SWB in a broad sense have rarely been examined in the *long run*. Consequently, our findings are difficult to compare with previous longitudinal studies and extend these studies.

Surprisingly, in our study intergenerational care did not affect any of the different outcome measures significantly. These findings support a recent study by Roth, Fredman and Haley [35] emphasizing that the beneficial effects of informal caregiving (e. g. meaning in life, rewarding) might be underreported and somewhat neglected. These beneficial effects might counterbalance the negative effects related to intergenerational caregiving (e. g. increased level of burden).

Contrary, intragenerational caregiving had a tremendous negative effect on mental health in our study and in recent studies [13–15]. This may be due to the intense care of a partner and the strong emotional closeness with the care-recipients [16] which might result in jeopardizing their own health by caring for their loved one [36]. Nevertheless, it is quite puzzling why intragenerational caregiving affected CWB in women, whereas it did not affect their mental health. Moreover and the other way around, spousal caregiving did not affect CWB in men, but affected their mental health. Further research is required to better understand the mechanisms involved.

### Strengths and limitations

This is the first longitudinal study examining the *long-term* effect of intra- and intergenerational caregiving on SWB in older adults in Germany. Furthermore, SWB was assessed by using validated instruments (e. g. SWLS). Additionally, by covering different aspects of SWB (AWB and CWB as well as functional and mental health), it was tested whether intra- and intergenerational caregiving affected the various dimensions of SWB differently.

In addition, time constant unobserved heterogeneity was taken into account by using FE regression, leading to consistent estimates under the assumption of strict exogeneity. Moreover, a population-based sample of community-dwelling individuals aged 40 and above living in Germany was used.

One limitation of this study is that short-term changes may be covered for reasons of adaptation processes [37] since time span between our waves was rather long. Furthermore, due to endogenous selection bias in the German Ageing Survey [38], our estimates might be somewhat biased downwards. Additionally, other predictors (e. g. need for care) may play a role in the relationship between informal caregiving and SWB [39].

## Conclusion

Our findings highlight the need for separating intra- from intergenerational caregiving. Moreover, our findings highlight the importance of intragenerational caregiving for mental health (men) and CWB (women). In terms of significance and magnitude particularly the former relation should be underlined. Consequently, interventions to avoid mental illness in men providing care for her (marital) partner are strongly needed.

Informal caregiving remains a complex phenomenon that can have both deleterious and beneficial effects [35, 40]. Due to this complexity, future longitudinal studies should try to disentangle the various effects of inter- and intragenerational caregiving (e. g. by including domain satisfactions as outcome variables and by including anticipation and adaptation effects [41]–as far as data are available).
